# The Mediating Roles of Attitude Toward COVID-19 Vaccination, Trust in Science and Trust in Government in the Relationship Between Anti-vaccine Conspiracy Beliefs and Vaccination Intention

**DOI:** 10.3389/fpsyg.2022.936917

**Published:** 2022-09-02

**Authors:** Miriam Capasso, Daniela Caso, Gregory D. Zimet

**Affiliations:** ^1^Department of Humanities, University of Naples Federico II, Naples, Italy; ^2^Department of Pediatrics, Indiana University School of Medicine, Indianapolis, IN, United States

**Keywords:** anti-vaccine conspiracy beliefs, attitude toward vaccination, COVID-19 vaccine, trust in science, trust in government

## Abstract

Since the outbreak of COVID-19, many conspiracy theories have spread widely, which has the potential to reduce adherence to recommended preventive measures. Specifically, anti-vaccine conspiracy beliefs can have a strong negative impact on COVID-19 vaccination attitude and intention. The present study aimed to clarify how such beliefs can reduce vaccination intention, exploring the possible mediating roles of attitude toward vaccination, trust in science, and trust in government, among a sample of 822 unvaccinated Italian adults (Women = 67.4%; *M*_age_ = 38.1). Path analysis showed that anti-vaccine conspiracy beliefs influenced intention to get vaccinated both directly and indirectly through the mediating effects of attitude, trust in science, and trust in government. In particular, the simple mediating effect of attitude was the strongest one, followed by the serial mediating effect of trust in science and attitude itself. Findings provide insights into the design of interventions aimed at reducing misinformation and subsequent vaccine hesitancy.

## Introduction

The rapid spread of the COVID-19 pandemic has faced countries worldwide with the challenge of promoting the adoption of effective behaviors to prevent both infection and disease. Since the approval of the first COVID-19 vaccines at the end of 2020 (i.e., Pfizer – BioNTech and Moderna vaccines), vaccination has represented the targeted behavior on which the government’s primary efforts have been and continue to be focused. In Italy, there was a high level of participation in the vaccination campaign, with about 79% of the population fully vaccinated one year after the official start of the vaccination campaign (Ritchie et al., 2020). However, despite these encouraging data, the number of unvaccinated remains worrying, with the lowest percentages of adherence to the vaccination campaign in younger age groups (e.g., under 40s; Italian Ministry of Health, 2022). Indeed, according to the latest data from the Italian National Institute of Health (2022), during the period from 01/02/2021 to 05/10/2021, there were 38,096 deaths from COVID-19, of which 88.3% were among unvaccinated people. To maximize vaccination uptake in all age groups and avoid both severe effects of the disease and mortality, it is crucial to identify which psychological factors can affect the intention to get vaccinated.

In the context of psychological literature exploring why some people decide to get vaccinated whereas others do not, there have been several studies showing that attitude represents one of the strongest predictors of vaccination intention (and consequent uptake) for both generic vaccinations (Askelson et al., 2010; Britt and Englebert, 2018; Cha and Kim, 2019) and COVID-19 vaccination (Capasso et al., 2021; Guidry et al., 2021; Paul et al., 2021). Attitude toward behavior refers to the favorable or unfavorable evaluation of the behavior in question (Ajzen and Fishbein, 1977), including both cognitive (i.e., getting vaccinated is useful, safe, and effective) and affective (i.e., getting vaccinated is enjoyable, satisfying and desirable) components. Recent studies (e.g., Xiao, 2021) revealed that cognitive evaluations about getting vaccinated are key determinants of vaccination intention, as the choice to get vaccinated is often linked to assessments of how safe (for example, in terms of possible short- and long-term side effects) or effective the vaccine is in preventing a certain disease (Hwang, 2020). Considering the strong predictive role of attitude on vaccination intention, it may be worth exploring which factors can shape such evaluations and, in turn, the intention to get vaccinated.

Attitude toward vaccination may be negatively affected by anti-vaccine conspiracy theories (Jolley and Douglas, 2014; Shapiro et al., 2016; Hornsey et al., 2018). In general, conspiracy theories try to explain particular events or situations by interpreting them as the result of the action of “strong powers” (i.e., conspirators; Jolley and Douglas, 2014) that are able to influence individual and collective decisions, coordinating with each other and acting in secret agreement (Mancosu et al., 2017). These theories cover a wide range of phenomena, including climate change, genetically modified organisms, terrorist attacks and wars, and include the alleged origins of the COVID-19 pandemic (van Prooijen and Jostmann, 2013; Pummerer et al., 2022). Although not based on concrete evidence and being consistently rejected by the scientific community, conspiracy theories continue to be widespread, mainly due to the uncontrollable proliferation of fake news on non-scientific websites and social networking sites (Allington et al., 2021). Popular conspiracy theories about COVID-19 vaccines propose, for example, that such vaccines contain microchips which would be used to obtain people’s biometric data and control humanity, are used to modify humans genetically, or could cause infertility (Islam et al., 2021; Sallam et al., 2021). In spite of the many studies devoted to the identification of psychological factors able to explain the tendency to conspiracy mentality (van Prooijen and Jostmann, 2013; van Prooijen, 2017; Furnham and Grover, 2021), not enough attention has been paid to the outcomes of vaccination conspiracy theories and, specifically, to the deepening of the processes by which they can affect the attitude toward vaccinating against COVID-19 and the consequent intention or, in other words, to the psychological variables that can mediate this relationship.

In this regard, several studies suggest that one of the major consequences of endorsing such beliefs may consist in a reduction of trust at various levels: for example, trust in science, health care institutions, or government (Freeman et al., 2020; Agley and Xiao, 2021; Simione et al., 2021). In the context of COVID-19 vaccine hesitancy, however, we believe it might be helpful to compare such different levels of trust and examine which one can best explain the relationship between anti-vaccine conspiracy beliefs and COVID-19 vaccination attitude and intention. The present study considers two specific aspects of trust: trust in science and government (specifically, in the government’s capabilities to deal with the pandemic). As regards the first aspect, conspiracy mentality is strongly associated with a rejection of science in general and distrust of specific scientific findings and discoveries, including those related to vaccines (Lewandowsky et al., 2015). Lack of trust in science and doubts about the research results on vaccines – for example, data on their safety and efficacy – may translate into reluctance to get vaccinated (Jolley and Douglas, 2014). In this regard, Milošević Ðorđević et al. (2021), investigating anti-vaccine behavior of Serbian adults, found that trust in science and healthcare institutions significantly mediated the relationship between the endorsement of vaccination conspiracy theories and intention to get vaccinated or vaccinate children. Although the authors evaluated general vaccination intentions (i.e., vaccinating against any vaccine-preventable disease), it is plausible to hypothesize that such relationships may be even stronger when it comes to the COVID-19 vaccines, considering the proven link between belief in conspiracies, trust, and COVID-19 vaccination intention (Soveri et al., 2021).

Along with trust in science, conspiracy beliefs can also negatively impact trust in government. In relation to this issue, findings from previous studies have indicated that believing in conspiracy theories may lead to a mistrust in government and key political institutions, which in turn could contribute to reducing the intention to get vaccinated. For example, Freeman et al. (2020) explored the associations between COVID-19 conspiracy beliefs, trust in different institutions (both medical and political), and compliance with government guidelines (e.g., washing hands, respecting social distancing, taking diagnostic testing, accepting the vaccine when available), on a sample of English adults during the first wave of the pandemic. The authors demonstrated that COVID-19 conspiracy beliefs were negatively associated not only with trust in doctors and scientists but also with political institutions, particularly the United Kingdom government. In addition, endorsing conspiracy beliefs was significantly related to lower self-reported adherence to government guidelines. However, the study was carried out prior to the approval of any vaccine against COVID-19 and did not evaluate the potential indirect effect of conspiracy theories on the considered behaviors, for example, *via* different levels of trust. Also, the authors only evaluated associations of COVID-19 conspiracy beliefs – but not anti-vaccine focused ones – with mistrust and following government guidelines. Instead, in a more recent study, McCarthy et al. (2022) compared the effect of three different conspiracy theories on trust in government and COVID-19 vaccine hesitancy (unwillingness to receive a vaccination for COVID-19). Testing three separate models, they found that the generic COVID-19 conspiracy beliefs (i.e., believing that COVID-19 is a biological weapon made by China) did not affect trust in government, unlike the “government”-specific (i.e., believing that the government is using COVID-19 to limit people’s freedom) and “vaccine”-specific (i.e., believing the vaccine is a tool to harm or control people) conspiracy theories.

With respect to the link between trust and vaccination attitude and intention, several studies (Yaqub et al., 2014; Dubé and Gagnon, 2018; Badur et al., 2020) have proven that lack of trust (in science and government) is associated with hesitant attitudes toward vaccination. In this regard, Badur et al. (2020) argued that mistrust might lead people to question the safety and efficacy of vaccines and, consequently, delay or refuse vaccination. In support of this argument, a review by Yaqub et al. (2014) reported that lack of trust in the most influential sources of vaccination information (e.g., doctors, pharmaceutical companies, and government) is the main reason for holding negative vaccination attitudes. These findings have also been supported in the context of vaccination against COVID-19 (Latkin et al., 2021; Paul et al., 2021; Trent et al., 2022). More specifically, in a study on 32 countries (de Figueiredo and Larson, 2021), it emerged that believing that the government is handling the pandemic well was associated with higher vaccination acceptance in most examined countries. Moreover, a recent study by Caso et al. (2021) has demonstrated that trust can indirectly reduce vaccination intention by shaping the general attitude toward the vaccination in question. This result is consistent with the above-mentioned research works (Capasso et al., 2021; Guidry et al., 2021; Paul et al., 2021), showing that attitude represents one of the strongest and more proximal predictors of vaccination intention and could further explain the findings of those studies in which no direct impact of trust on hesitancy emerged, especially when the latter has been operationalized as an intention rather than a global attitude toward vaccination (see McCarthy et al., 2022).

**FIGURE 1:**
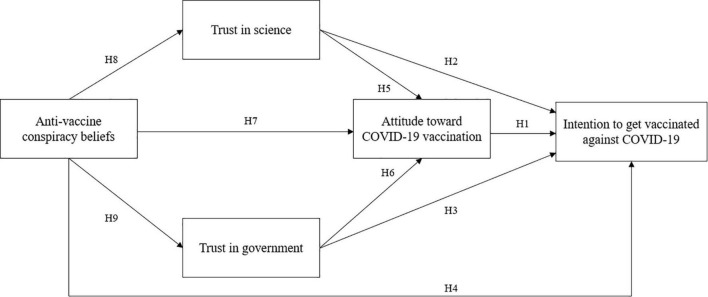
Hypothesized model.

Overall, many studies have been conducted on the links between anti-vaccine conspiracy theories, trust, attitude, and vaccination intentions, but, as far as we know, none of them has currently compared the mediating roles of trust in science and government, also considering their potential indirect impact on intention *via* attitude. Thus, taking into account the above-mentioned line of reasoning, we hypothesized a model which may clarify the relationship between anti-vaccine conspiracy beliefs and intention to receive the COVID-19 vaccine (Figure 1). Specifically, we hypothesized that intention would be positively predicted by attitude (Hypothesis 1 — H1), trust in science (Hypothesis 2 — H2), and trust in government (Hypothesis 3 — H3). Conversely, we expected that anti-vaccine conspiracy beliefs would negatively affect intention (Hypothesis 4 — H4). Likewise, we hypothesized that attitude would be positively predicted by trust in science (Hypothesis 5 — H5) and trust in government (Hypothesis 6 — H6) and negatively influenced by anti-vaccine conspiracy beliefs (Hypothesis 7 — H7). Moreover, we expected anti-vaccine conspiracy beliefs would negatively affect both trust in science (Hypothesis 8 — H8) and in government (Hypothesis 9 — H9). Lastly, in order to understand which mechanisms can better explain *how* anti-vaccine conspiracy beliefs eventually decrease the intention to get vaccinated, we explored whether this relationship was mediated by attitude, trust in science, or trust in government (Research Question 1 — RQ1), testing both simple and serial mediating effects.

## Materials and Methods

### Procedure and Participants

The current cross-sectional study used a questionnaire created through the Google Form online survey platform. The questionnaire was in the Italian language; thus, participants were recruited by advertising the questionnaire link on some of the main Italian social networking sites (e.g., Facebook groups and Instagram pages). In order to be eligible take part in this study, participants were required (1) to be of legal age (age >18) and (2) not yet vaccinated against COVID-19.

We carried out *a priori* power analysis to estimate the required sample size for detecting a small effect size (*f*^2^ = 0.02) for multiple regression analysis with four predictors, an alpha = 0.05 and power = 0.80. The estimated sample size was *N* = 602. Thus, we planned to recruit *N* >602 into the study in order to achieve more than sufficient power to detect additional mediation effects, also taking into account a 20% dropout due to ineligibility. Among the invited participants, 822 met the inclusion criteria and completed the questionnaire after being informed of the anonymity of the data collection and giving informed consent.

Respondents, all Italians, were mainly women (67.4%) aged 18 through 78 years (*M* = 38.1; *SD* = 14.7). The majority were married or in a romantic relationship (64.8%), had a high school diploma or a degree (84.2%), and reported being in a middle socio-economic status (74.3%). Regarding political orientation, most people were apolitical or left-wing (35 and 38%, respectively). Finally, in relation to religious orientation, most of the participants (66%) declared themselves Catholic. Concerning experience with COVID-19, the overwhelming majority (90.6%) never tested positive for COVID-19.

Data were collected between March and May 2021.

### Measures

In the first section of the questionnaire, participants filled out the informed consent form. Thereafter, they were instructed to answer all subsequent questions by thinking specifically about the COVID-19 vaccine. This indication was motivated by the fact that some measures were generic and not focused on the COVID-19 vaccine, particularly the anti-vaccine conspiracy beliefs scale (see the limitation section in the Discussion). Then, the following measures were administered in the same order to all the participants.

*Demographic variables*. We collected information about participants’ socio-demographic characteristics, i.e., age, gender, socio-economic status, marital status, education, political orientation, and religious orientation. We also asked if they had ever tested positive for COVID-19.

*Intention to get vaccinated against COVID-19* was measured with three items (e.g., “*I intend to get vaccinated against COVID-19*”; adapted from Askelson et al. (2010)). Participants indicated their agreement with the items on a Likert scale ranging from *completely disagree* (1) to *completely agree* (5). Cronbach’s α = 0.96.

*Attitude toward COVID-19 vaccination* was measured with 4 items using a semantic differential scale ranging from 1 (negative pole) to 5 (positive pole). Participants were asked to evaluate whether “Vaccinating against COVID-19” would be: *harmful/beneficial*, *useless/useful*, *dangerous/safe*, *irresponsible/responsible*. Higher scores indicated a more favorable attitude toward vaccination. Cronbach’s α = 0.91.

*Trust in science* was assessed using the *Belief in science scale* (Farias et al., 2013). The scale consisted of 10 items evaluating a general belief in science and acceptance of the scientific method. Participants were asked to indicate their degree of agreement with the items on a Likert scale ranging from *strongly disagree* (1) to *strongly agree* (6). A sample item is “*We can only rationally believe in what is scientifically provable*”. Cronbach’s α = 0.92.

*Trust in government* was assessed through 4 items (e.g., “*Do you think the authorities are doing a good job in dealing with the COVID-19 pandemic?*”; adapted from Prati et al. (2011)) using a scale ranging from *not at all* (1) to *very much* (5). In this study, trust in government is defined as the trust in the government’s capabilities to manage the pandemic. In the following sections, we will label such variable as “trust in government” for brevity. Cronbach’s α = 0.81.

*Anti-vaccine conspiracy beliefs* were assessed by adapting the 8-item *Anti-vaccine conspiracy belief scale* (Jolley and Douglas, 2014). Participants were asked to indicate their degree of agreement with the items on a Likert scale ranging from *strongly disagree* (1) to *strongly agree* (7). A sample item is “*Immunizations allow governments to track and control people*” (Cronbach’s α = 0.89).

### Data Analysis

Statistical analyses were conducted using R version 3.6.3 statistical software. Descriptive statistics were used to describe the participants’ socio-demographic and psychological characteristics. To test our hypothesized model, we carried out a *path analysis* using the R package lavaan (Rosseel, 2012). Since our data were not normally distributed (Skewness and Kurtosis values >| 1| for intention, attitude and anti-vaccine conspiracy beliefs variables), we estimated the parameters using the maximum likelihood estimation with robust standard errors and a Satorra-Bentler scaled test statistic (“MLM” estimator in lavaan). Indeed, MLM allows obtaining the goodness-of-fit statistics in the case of violation of assumptions of normality, computing standard errors and a mean-adjusted chi-square test statistic that are robust to non-normality (Maydeu-Olivares, 2017).

Effects of the considered predictors (anti-vaccine conspiracy beliefs, trust in science, trust in government) on our main dependent variables (attitude toward COVID-19 vaccination and intention to get vaccinated) were estimated by controlling for past COVID-19 positivity, socio-economic status, and education level, as past studies (e.g., Wong et al., 2020) indicated that such variables may affect vaccination attitude and/or intention.

Additionally, using the Monte Carlo method (Preacher and Selig, 2012), we tested the following mediating effects in the relationship between anti-vaccine conspiracy beliefs and intention: (a) simple mediating effect of attitude; (b) simple mediating effect of trust in science; (c) simple mediating effect of trust in government; (d) serial mediating effect of trust in science and attitude; and (e) serial mediating effect of trust in government and attitude. We estimated each mediating effect by running 20,000 repetitions to get a 95% confidence interval. Indirect effects were considered statistically significant if the confidence intervals (CIs) did not include zero. Where appropriate, we also carried out pairwise contrasts of indirect effects to identify the strongest ones. The goodness of fit was evaluated using the following indices: Chi-square test (χ^2^), RMSEA (Root Mean Square Error of Approximation), CFI (Comparative Fit Index), TLI (Tucker-Lewis Index) and SRMR (Standardized Root Mean Square Residual). The fit can be considered adequate with a non-significant Chi-square, CFI and TLI values of at least 0.90, and RMSEA and SRMR values lower than 0.06 and 0.08, respectively (Hu and Bentler, 1999). However, the Chi-square test is influenced by the sample size and tends to be significant with very large samples even when there are no differences (Bentler and Bonett, 1980). For this reason, it is appropriate to refer to the other fit indices. All the answers to the questionnaire were mandatory, so there were no missing values.

## Results

### Descriptive Statistics

As for the psychological variables (Table 1), results showed that, on average, participants reported a very high level of intention to get vaccinated, a strong positive attitude toward COVID-19 vaccination, quite high levels of trust in science, moderate levels of trust in government and low anti-vaccine conspiracy beliefs. Moreover, all the correlations between the variables were statistically significant: in particular, intention, attitude, trust in science, and trust in government positively correlated with each other and were, in turn, negatively associated with anti-vaccine conspiracy beliefs.

**TABLE 1:** Descriptive statistics and Pearson’s correlations among the psychological variables.

Variable	*M*	*SD*	*Range*	1.	2.	3.	4.	5.
1. Intention to get vaccinated against COVID-19	4.32	1.00	1 – 5	1				
2. Attitude toward COVID-19 vaccination	4.28	0.83	1 – 5	0.76**	1			
3. Trust in science	4.39	0.98	1 – 6	0.33**	0.36**	1		
4. Trust in government	2.26	0.70	1 – 5	0.18**	0.19**	0.10*	1	
5. Anti-vaccine conspiracy beliefs	2.07	1.12	1 – 7	–0.55**	–0.58**	–0.34**	–0.10*	1

*p < 0.05; **p < 0.01.

### Path Model

**FIGURE 2:**
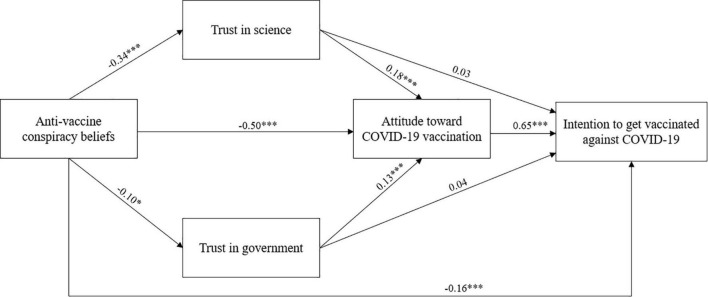
Path analysis model with standardized regression coefficients. *p < .05; ***p < .001.

Regarding the hypothesized relationships, the model provided a good fit to the data, with χ^2^ = 38.417, df = 7, *p* < 0.001; CFI = 0.975; TLI = 0.923; RMSEA = 0.074; SRMR = 0.035. Results showed that almost all the hypotheses were confirmed (Figure 2). Specifically, intention to get vaccinated against COVID-19 (control variables^1^ effects on intention: past COVID-19 positivity: β = −0.02, *p* = 0.43; socio-economic status: β = 0.02, *p* = 0.41; education level: β = 0.03, *p* = 0.43) was positively predicted by attitude toward COVID-19 vaccination and negatively by anti-vaccine conspiracy beliefs, confirming H1 and H4. Conversely, the direct effects of trust in science and government on intention were not significant; thus, H2 and H3 were not supported. Moreover, consistent with H5, H6, and H7, attitude (control variables effects on attitude: past COVID-19 positivity: β = 0.05, *p* = 0.16; socio-economic status: β = 0.00, *p* = 0.96; education level: β = 0.02, *p* = 0.53) was positively affected by trust in science and trust in government, and negatively by anti-vaccine conspiracy beliefs. The latter negatively predicted both trust in science and trust in government, confirming H8 and H9.

### Mediation Analyses

In order to answer RQ1, we ran two serial mediation analyses. In the first analysis, we tested the serial mediating effect of (1) trust in science and (2) attitude in the relationship between anti-vaccine conspiracy beliefs and intention to get vaccinated against COVID-19. To do so, we first verified the simple mediating effects of both mediators. Results showed a simple mediation by attitude (*Indirect effect* = −0.289; 95% CI [−0.357, −0.225]), but not by trust in science (*Indirect effect* = −0.010; 95% CI [−0.028, 0.006]). Nevertheless, a serial mediating effect of trust in science and attitude was supported (*Indirect effect* = −0.035; 95% CI [−0.050, −0.021]). In the second analysis, we instead tested the serial mediating effect of 1) trust in government and 2) attitude. Results did not support a simple mediation effect of trust in government (*Indirect effect* = −0.003; 95% CI [−0.000, 0.000]), but showed a weak serial mediating effect of trust in government and attitude (*Indirect effect* = −0.010; 95% CI [−0.013, −0.001]). Moreover, pairwise contrasts of indirect effects indicated that the simple mediating effect of attitude was stronger than both the serial mediating effect of trust in science and attitude (*Effect* = −0.254; 95% CI [−0.323, −0.192]) and the serial mediating effect of trust in government and attitude (*Effect* = −0.283; 95% [CI −0.350, −0.220]). Finally, results also supported a significant difference between the two examined serial mediating effects (*Effect* = −0.028; 95% CI [−0.044, −0.014]).

## Discussion

The present study aimed to test the direct and indirect effects of anti-vaccine conspiracy beliefs on intention to get vaccinated against COVID-19. Indeed, the pandemic, like all events that are threatening, uncertain, and difficult to understand (van Prooijen and Jostmann, 2013; Caso et al., 2022), has represented fertile ground for the development or strengthening of conspiracy theories linked to both the origins of the virus and vaccines (Lynas, 2020; Eberhardt and Ling, 2021; Pummerer et al., 2022). While many of these beliefs are not harmful in themselves, it has been amply demonstrated how they can negatively impact vaccine acceptance (Jolley and Douglas, 2014), including COVID-19 vaccines (Soveri et al., 2021). This problem shows that it is important to carry out research into the mechanisms by which endorsing such theories can reduce COVID-19 vaccine intention, in order to identify the psychological variables that should be taken into account in future interventions aimed at counteracting COVID-19 vaccine hesitancy.

In line with our hypotheses, findings showed that belief in anti-vaccine conspiracy theories significantly affected intention to get vaccinated against COVID-19, both directly and indirectly, by decreasing trust in science, trust in government, and the global attitude toward the COVID-19 vaccination. Specifically, vaccination intention was positively predicted by attitude toward COVID-19 vaccination, which represented its strongest precursor, and negatively by anti-vaccine conspiracy beliefs, supporting H1 and H4. The strong relationship that emerged between vaccination intention and attitude is not surprising, considering the literature discussed above on the key role of attitude in predicting vaccination intentions, even in the context of traditional vaccines, for example, those that are mandatory or recommended for children and adolescents (Caso et al., 2019, 2021). Moreover, the negative association between anti-vaccine conspiracy theories and intention provides further evidence in support of a direct impact of these beliefs on willingness to vaccinate against COVID-19 (Milošević Ðorđević et al., 2021), confirming the need for research to focus on the specific impact of anti-vaccine conspiracy beliefs, rather than those related to the pandemic, which may not be relevant to people’s intention to get vaccinated (Yang et al., 2021).

Contrary to what was hypothesized (H2 and H3), however, intention was not significantly predicted either by trust in science or government. On the other hand, consistent with H5 and H6, both types of trust significantly affected attitude toward COVID-19 vaccination. This finding aligns with the results of previous studies (Caso et al., 2021), confirming that trust is strongly related to the global attitude toward vaccinating and vaccines rather than being a proximal predictor of intention. In turn, both the attitude and the two types of trust were significantly influenced by anti-vaccine conspiracy beliefs, thus confirming H7, H8, and H9. In the first place, it turned out that the more people endorsed anti-vaccine conspiracy beliefs, the weaker was their trust in science and government. These results are consistent with recent studies showing the negative impact of conspiracy mentality on trust in the official sources of information on the vaccine, both medical and political (McCarthy et al., 2022; Milošević Ðorđević et al., 2021; Soveri et al., 2021; Zhang et al., 2021). As highlighted by Simione et al. (2021), such a mechanism could reflect the tendency of these beliefs to feed themselves by disregarding information that could question or deny them, a bias often called motivated reasoning. Furthermore, it is possible to speculate that a lack of confidence in science or policymakers goes hand in hand with a propensity to obtain information through unofficial sources (e.g., the Internet and social networking sites; Caso, 2015) increasing, in a sort of chain reaction, the risk of coming across unreliable information about the vaccine (Dubé and Gagnon, 2018).

Looking at regression coefficients, the results seem to suggest a stronger impact of anti-vaccine beliefs on trust in science than trust in government. However, this interpretation should be taken with caution, as this data could reflect differences in internal consistencies of the two measures (i.e., trust in government showed a lower Cronbach’s α than that of trust in science). Nevertheless, the negative influence of these beliefs raises concerns in both cases. In fact, the latter seem to have the power to erode trust in science, in the work of scientists, and in the reliability of the results of their discoveries. Unfortunately, such trust appears particularly relevant in the context of the COVID-19 vaccination, considering the record time in which it was developed and approved and the consequent widespread doubts and concerns among the population (Rosenthal and Cummings, 2021). These concerns, in turn, are often intensified rather than contained by the proliferation of unsubstantiated speculations. As for trust in government, our results are in line with studies on past pandemics (e.g., influenza H1N1 outbreak; Prati et al., 2011; Chuang et al., 2015), showing that mistrust in the government’s competence in dealing with the pandemic, although not necessarily translating into a lack of adhesion to basic recommended behaviors (for example, washing hands or maintaining social distancing), negatively predicts vaccine acceptance. This evidence highlights the need to build and maintain trust and establish effective communication about public health before pandemics occur (Chuang et al., 2015).

Over and above the effect of anti-vaccine conspiracy beliefs on trust, results showed that the strongest negative effect linked to the endorsement of these beliefs was related to the worsening of attitude toward the COVID-19 vaccination. Such a result adds substantial evidence to the fact that the more people agree with them, the more unfavorable the vaccine evaluation becomes, such that people start doubting whether vaccination is safe, effective, or beneficial (Jolley and Douglas, 2014). Moreover, in response to our RQ1 about which mechanisms can better explain how anti-vaccine conspiracy beliefs eventually decrease the intention to get vaccinated, the mediation analyses showed that the strongest mediating effect in the relationship between anti-vaccine conspiracy beliefs and intention was the simple effect of attitude. These findings prompt important considerations. First, the evidence that these beliefs act on intention primarily by worsening attitude suggests that interventions aimed at containing the impact of misinformation on vaccine intentions should work on challenging all vaccine-related negative evaluations to which these beliefs lead, targeting their different facets. As highlighted in a recent review by Brewer (2021), working on attitudes can have an indirect effect on vaccine uptake by contributing to the creation of a climate of trust that can support vaccination acceptance. However, the other two identified mediation mechanisms (i.e., serial mediating effects of trust in science/government and attitude) suggest that efforts to improve attitudes toward COVID-19 vaccines – especially considering that people with high levels of anti-vaccine conspiracy beliefs can be resistant to health messaging because of distrust – may be pointless without parallel work aimed at building trust in science and government to reduce or counterbalance the negative effect of anti-vaccine conspiracy beliefs. Effective strategies to retain or regain trust in science include enhancing people’s perceptions that scientists have knowledge and expertise, are honest and caring, and provide clear information (Roberts et al., 2013). Likewise, to increase trust in government, political decisions should be perceived by the population as fair, transparent, and effective (McCarthy et al., 2022). This could serve to address the rumors of vaccination conspiracy theories before they become widespread, given the difficulty in disconfirming them once well established (Soveri et al., 2021). Finally, using such strategies to combat misinformation and medical distrust is also crucial to promoting COVID-19 vaccination of children and adolescents (Zimet et al., 2021), considering the stronger vaccine hesitancy which can characterize parental choice (Szilagyi et al., 2021).

Clearly, there are some limitations to the current study. First, its cross-sectional nature limits any possible causal claims. Second, the use of self-report measures – as is the case in most of the studies in this research area – may have led the participants to answer the questions in a socially desirable way, limiting the validity of the results. Third, since we relied on a non-probabilistic sampling, we cannot argue that the results are generalizable to the Italian population. Fourth, at the time of study design, we could not find any instrument for the measurement of the COVID-19 vaccine-specific conspiracy beliefs; thus, we relied on a generic anti-vaccine conspiracy belief scale (Jolley and Douglas, 2014). However, despite most of the conspiracy beliefs investigated by this scale are also applicable to the COVID-19 vaccines (i.e., beliefs that vaccines contain microchips or that vaccinations allow governments to track and control people; Islam et al., 2021; Sallam et al., 2021), we are aware that such instrument may not have grasped some other specific beliefs (e.g., conviction that the vaccine causes infertility) and, in general, that conspiracy theories may have changed by the time of the study. Also, it cannot be excluded entirely that even a small percentage of participants may have had different vaccines in mind in answering items on anti-vaccine conspiracy beliefs, despite the initial indication given in the questionnaire to think specifically about the COVID-19 vaccine. To eliminate such a bias, future research should use specific measures focused on the COVID-19 vaccine conspiracy theories. Besides, we recognize that we have investigated a sample of participants who, on average, showed low levels of anti-vaccine conspiracy beliefs and that, therefore, our results need to be confirmed by future studies focused on individuals endorsing moderate or high levels of such beliefs. Finally, we cannot rule out that the relationships investigated in the present study are someway bidirectional (e.g., low levels of trust affect high levels of conspiracy beliefs) or that there are other possible mediators that we have not included in the model. Thus, further studies would benefit from investigating the existence of a possible cause-and-effect relationship between conspiracy beliefs and trust or vice versa (e.g., through experimental studies), evaluating more specific nuances of trust which take into account the different actors involved (e.g., scientists, general practitioners, health professionals, international and national policymakers) in vaccination programs.

## Conclusion

The results of the present study shed light on the psychological mechanisms by which anti-vaccine conspiracy beliefs can undermine attitude toward COVID-19 vaccination and the consequent intention to get vaccinated. Specifically, conspiracy beliefs are suggested to work first by deteriorating attitudes and, secondly, by destroying trust in official sources of information about COVID-19, i.e., science, scientists, and political institutions. Therefore, parallel action is needed on two fronts. On the one hand, a greater information and communication effort is needed to improve people’s attitude toward COVID-19 vaccination, with particular attention to the characteristics of efficacy and safety, which seem to represent the key aspects underlying a favorable evaluation of such vaccine (Capasso et al., 2021). On the other hand, in a complementary way, regaining social trust by the population can, to an extent, serve itself as a “vaccine” against the effects of conspiracy beliefs; in fact, if it seems plausible that these beliefs reduce the trust, the opposite could also be true, i.e., having high levels of trust can, in turn, protect against the danger of disinformation (Šrol et al., 2021). Hence, as highlighted by Dubé and Gagnon (2018), it is not just a question of increasing trust in the specific vaccine (the *product*), but also and above all in the political institutions (the *policymakers*) and health services (the *providers*) that recommend, promote, and govern vaccination programs. Even more so in the context of the COVID-19 vaccination, achieving this goal requires synergistic work by the health and political authorities.

## Data Availability Statement

The raw data supporting the conclusions of this article will be made available by the authors, without undue reservation.

## Ethics Statement

The studies involving human participants were reviewed and approved by the Ethics Committee of Psychological Research of the University of Naples Federico II. The patients/participants provided their written informed consent to participate in this study.

## Author Contributions

MC and DC conceived and designed the study and wrote the first draft of the manuscript. MC organized the database and performed the statistical analysis. GZ collaborated to the study design and revised the manuscript. All authors contributed to the manuscript revision, read, and approved the submitted version.

## Conflict of Interest

GZ has served as a consultant to Merck related to HPV vaccination and as an external advisory board member for Moderna (related to COVID-19 vaccination) and Pfizer (related to meningococcal vaccination). He is also an investigator on research funded by Merck’s Investigator Studies Program, administered through Indiana University. The remaining authors declare that the research was conducted in the absence of any commercial or financial relationships that could be construed as a potential conflict of interest.

## Publisher’s Note

All claims expressed in this article are solely those of the authors and do not necessarily represent those of their affiliated organizations, or those of the publisher, the editors and the reviewers. Any product that may be evaluated in this article, or claim that may be made by its manufacturer, is not guaranteed or endorsed by the publisher.

## Footnotes

1 We recoded control variables into dummy variables for analysis purposes (past COVID-19 positivity: yes = 1, no = 0; socio-economic status: middle and high = 1, low = 0; education level: high school diploma or higher qualification = 1, compulsory education = 0).
